# Preparation and characterization of a GSH-responsive drug-loaded polymer nanoparticle/silk fibroin composite hydrogel

**DOI:** 10.3389/fbioe.2025.1643800

**Published:** 2025-07-03

**Authors:** Yanjiu Li, Rong Li

**Affiliations:** College of Chemistry and Chemical Engineering, Donghua University, Shanghai, China

**Keywords:** GSH-responsive nanoparticles, silk fibroin hydrogel, curcumin delivery, composite hydrogel system, microenvironment-responsive drug release

## Abstract

This study addressed the poor water solubility and low bioavailability of curcumin (CUR), along with the inadequate stability of existing nano-delivery systems. A composite delivery system (GEL-PNES-CUR) combining a glutathione (GSH)-responsive drug-loaded polymeric nanoparticle (NP@PNES-CUR) with a silk fibroin hydrogel (SF-GEL) was constructed. Firstly, SF-GEL was prepared using the HRP/H_2_O_2_ system. A three-dimensional porous network structure was imparted to SF-GEL through enzymatic cross-linking. SF-GEL exhibited injectability, making it suitable for minimally invasive therapy, combined with a high swelling ratio (546%) and excellent rheological properties (storage modulus G′ reaching 10,000 Pa). Secondly, a GSH-sensitive polymer (PNES) was designed and synthesized. Rigid bipyridyl groups were introduced into the PNES backbone to enhance π-π stacking interactions. Subsequently, PNES was combined with CUR to prepare NP@PNES-CUR.For NP@PNES-CUR, an encapsulation efficiency of 47.7% for CUR was achieved (compared to only 7.5% for the control group). The GSH-responsive characteristics of disulfide bonds were utilized to achieve CUR release triggered by the inflammatory microenvironment (a release rate of 79.8% was reached within 24 h). Finally, NP@PNES-CUR was loaded into SF-GEL to prepare GEL-PNES-CUR. GEL-PNES-CUR was demonstrated to possess the function of sustained CUR release. Cell experiments indicated that GEL-PNES-CUR possessed good biocompatibility (cell viability >70%). This study provides a novel material for complex inflammation treatment, combining mechanical adaptability with controllable drug release functionality.

## 1 Introduction

Curcumin (CUR), as a natural anti-inflammatory drug, has garnered significant attention due to its broad pharmacological activities. However, the extremely low water solubility and bioavailability of CUR severely limit its clinical applications (Nelson et al., 2017). Although nano-delivery systems can improve the solubility of CUR, they face challenges such as non-specific distribution, inadequate stability *in vivo*, and mechanical properties mismatched with the dynamic physiological environment (Hoelder et al., 2012; Mollaei et al., 2021). For instance, inflammatory lesions like osteoarthritis or traumatic brain injury are often accompanied by complex mechanical environments, where existing nanocarriers struggle to maintain structural integrity and controlled drug release functionality under dynamic stress (Sharma, 2021; Feng et al., 2022).

**FIGURE 1:**
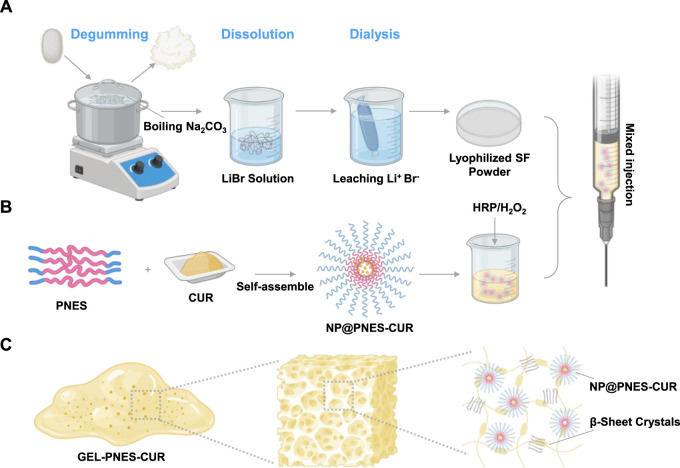
Schematic diagram of the preparation and structure of GEL-PNES-CUR. **(A)** Preparation process of lyophilized SF powder. **(B)** Preparation process of NP@PNES-CUR. **(C)** Structural schematic diagram of GEL-PNES-CUR.

Silk fibroin hydrogel (SF-GEL), owing to its excellent biocompatibility, injectability, and tunable mechanical properties, has emerged as an ideal choice for drug delivery carriers (Wang et al., 2008; Laomeephol et al., 2020). However, a single SF-GEL system is difficult to achieve precise drug release responsive to the inflammatory microenvironment. Based on this, a multifunctional composite delivery system combining a glutathione (GSH)-responsive drug-loaded nanoparticle (NP@PNES-CUR) with SF-GEL was constructed. This system possesses both mechanical support function under dynamic stress and controllable sustained drug release functionality (Wang Y. et al., 2025). Firstly, lyophilized SF powder was prepared through degumming, dissolution, dialysis, and lyophilization (Figure 1A). Secondly, to confer drug-specific release functionality to SF-GEL, the GSH-responsive polymer PNES was synthesized. CUR was encapsulated using PNES to prepare the GSH-responsive nano-drug NP@PNES-CUR. Finally, NP@PNES-CUR was loaded into a silk fibroin aqueous solution (SF-aq) obtained by reconstituting the lyophilized SF powder (Figure 1B). Gelation was then performed using the HRP/H_2_O_2_ system to prepare the SF-GEL composite material (GEL-PNES-CUR), which enables GSH-responsive sustained release of CUR, with its structure shown in Figure 1C.

Consequently, the innovations of this study lie in: (1) Significantly enhancing the drug encapsulation efficiency of the nanoparticles by introducing rigid bipyridyl groups into PNES to strengthen π-π stacking interactions; (2) Achieving CUR release triggered by the inflammatory microenvironment utilizing the GSH-responsive characteristics of disulfide bonds; (3) Providing a novel multifunctional material for complex inflammation treatment by combining the mechanically tunable properties of SF-GEL with the GSH-responsive CUR release capability of the nanoparticles.

## 2 Materials and methods

### 2.1 Preparation of SF-GEL

Silkworm cocoons were selected and immersed in an aqueous Na_2_CO_3_ solution. Degumming was performed in a boiling water bath for 30 min. After degumming, the material was washed three times with deionized water and dried. The dried material was then immersed in a LiBr solution and maintained at 60°C ± 2°C for dissolution over 4 h. The obtained solution was dialyzed for 72 h, filtered under suction, and lyophilized to obtain lyophilized SF powder.

Lyophilized SF powder was reconstituted to obtain SF-aq. SF-aq was then blended with aqueous solutions containing H_2_O_2_ (0.1 mM) and HRP (6 mg/mL). Gelation was carried out at 60°C to obtain SF-GEL.

### 2.2 Characterization of SF-GEL

SF-GEL was lyophilized and rapidly frozen in liquid nitrogen. Representative cross-sectional slices were fixed onto conductive adhesive tape surfaces. Conductive coating was applied by gold sputtering to enhance conductivity. Finally, the cross-section of the lyophilized cryogel was observed and analyzed by SEM.

SF-GEL samples were continuously soaked in PBS (pH = 7.4) at a constant temperature of 37 °C. Samples were taken at 1, 2, 3, 6, 12, 24, 36, and 48 h. Surface moisture was removed using filter paper, and the weight was recorded as W_s_. SF-GEL was then lyophilized for 72 h, and the weight after lyophilization was recorded as W_d_. The swelling ratio of SF-GEL is (W_s_-W_d_)/W_d_, and a time-swelling ratio curve was plotted.

Rheological characterization of SF-GEL was performed using an advanced rheometer ARES-G2 (TA Instruments, New Castle, DE). Testing was initiated after *in situ* gelation at 60 °C for 15 min.

The rheological properties of SF-GEL was determined by strain sweep testing. The loading gap was set to 50.0 mm, and the trimming gap was set to 0.1 mm. After cooling to 25°C, a 10 s wait period was applied. The frequency was set to 6.28 rad/s, and the strain sweep range was set from 0.01% to 100.0%, with ten points per decade. The relationship between G′, G″ and strain was obtained. A suitable maximum strain value within the rheological properties before the yield point was identified for use as the strain value in subsequent dynamic frequency sweep and time sweep tests. For dynamic frequency sweep, the parameters remained unchanged, with strain set to 0.1%. The relationship between G′, G″ and frequency for SF-GEL was tested. For time sweep, the parameters remained unchanged, with strain set to 0.1%. The relationship between G′, G″ and time for SF-GEL was tested.

### 2.3 Synthesis and characterization of PES and PNES

Ethyl (S)-2,6-diisocyanatohexanoate (Linker) and 2,2′-Dithiodiethanol were dissolved in N,N-dimethylformamide (DMF). After reaction at room temperature for 24 h, polyethylene glycol (mPEG_5000_) was added. The reaction temperature was increased until complete dissolution of mPEG_5000_ was achieved, and stirring was continued for an additional 24 h. Upon reaction completion, dialysis was performed, followed by lyophilization to obtain PES.

Ethyl (S)-2,6-diisocyanatohexanoate (Linker) and 2,2′-Dithiodiethanol were dissolved in N,N-dimethylformamide (DMF). 5,5′-bis(hydroxymethyl)-2,2′-bipyridine dissolved in super-dry DMF was added. After reaction at room temperature for 24 h, polyethylene glycol (mPEG_5000_) was added. The reaction temperature was increased until complete dissolution of mPEG_5000_ was achieved, and stirring was continued for an additional 24 h. Upon reaction completion, dialysis was performed, followed by lyophilization to obtain PNES.

Structural characterization of the product PNES was conducted using ^1^H NMR with deuterated dimethyl sulfoxide (DMSO-d_6_) as the solvent. The weight-average molecular weight of polymer PNES was analyzed by gel permeation chromatography (GPC). Fourier-transform infrared spectroscopy (FTIR) was employed to characterize the chemical structure of PNES, with the spectral measurement range set between 4,000 and 400 cm^-1^.

### 2.4 Characterization of NP@PES-CUR and NP@PNES-CUR

The particle size and zeta potential of NP@PES-CUR and NP@PNES-CUR were characterized using a Malvern Nano ZS90 dynamic light scattering (DLS) instrument. The measurement temperature was set at 25°C with a 120-s equilibrium time. Zeta potential and polydispersity index (PDI) were calculated using the cumulative analysis method. To assess the storage stability of NP@PNES-CUR, the DLS test was repeated daily for 5 days.

The morphology of NP@PNES-CUR was observed using transmission electron microscopy (TEM).

### 2.5 Drug release and characterization of NP@PNES-CUR

For the experimental group, GSH was added, while the control group maintained a PBS system. A 5 mL sample of NP@PNES-CUR was placed in dialysis bags for both groups, and samples were taken at six time points: 1, 2, 3, 6, 12, and 24 h.

The CUR concentration in the obtained samples was characterized using high-performance liquid chromatography (HPLC). The concentration of CUR at each time point was quantified by constructing a standard curve. The measured data were converted to cumulative release percentages, and a time-release curve was plotted.

The morphology of NP@PNES-CUR after dissociation was observed using transmission electron microscopy (TEM).

The particle size of NP@PNES-CUR after disintegration was measured using the same method employed for particle size characterization.

### 2.6 Preparation of GEL-PNES-CUR

GEL-PNES-CUR was prepared by blending SF-aq with NP@PNES-CUR and an HRP/H_2_O_2_ system, followed by gelation.

### 2.7 Characterization of GEL-PNES-CUR

GEL-PNES-CUR was prepared into pieces with dimensions of 20 × 10 × 5 mm^3^ and placed in dialysis bags. The samples were maintained at room temperature in a brown glass bottle and subjected to continuous shaking at 150 rpm. Samples were taken on days 2, 3, 5, 7, 9, and 11.

The CUR concentration in the obtained samples was characterized using HPLC, and a time-release curve was plotted.

Rat fibroblast cells (Rat-FB), derived from rat connective tissue, were selected as the cell model for this study. These cells were cultured in RPMI-1640/DMEM medium containing 10% fetal bovine serum (FBS) and 1% penicillin/streptomycin (P/S).

GEL-PNES-CUR and SF-GEL were seeded in 96-well plates, with a PBS group serving as the blank control. Rat-FB cells were then seeded onto the plates. The day of cell seeding was designated as day 0, and samples were collected on days 1, 3, and five to conclude the culture.

After the culture period, the culture medium was removed, and 100 μL of MTT solution (5 mg/mL) was added to each well. The plates were incubated at 37°C in a 5% CO_2_ incubator for 4 h. The medium was aspirated, and 150 μL of SDS solution was added to dissolve the formazan crystals. Absorbance was measured at 570 nm and 650 nm using a microplate reader. The final result was calculated by subtracting the absorbance at 650 nm from the absorbance at 570 nm.

## 3 Results and discussion

### 3.1 Preparation and characterization of SF-GEL

**FIGURE 2:**
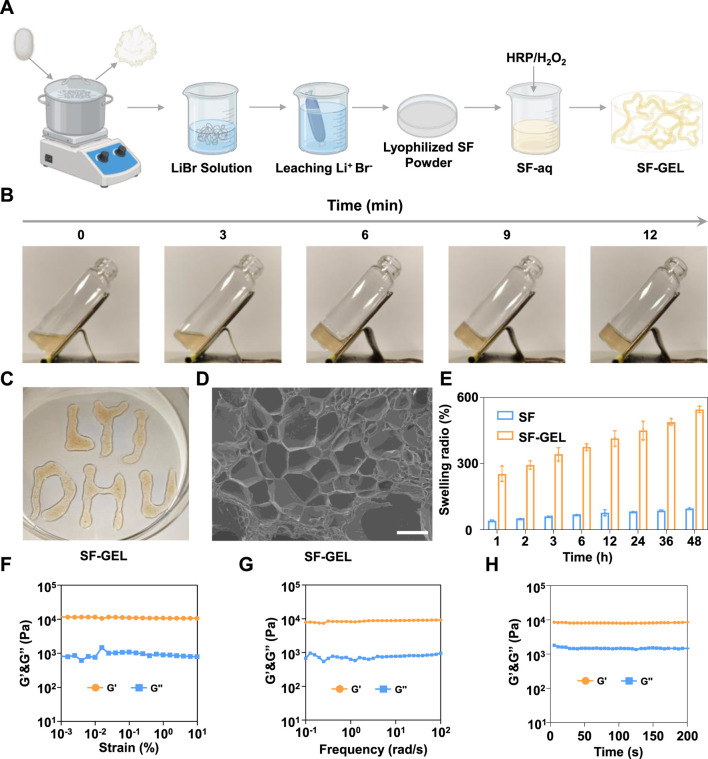
Gelation and performance characterization of SF-GEL. **(A)** Schematic diagram of the SF-GEL preparation process. **(B)** Gelation time of SF-GEL. **(C)** Injectability of SF-GEL. **(D)** SEM image of the SF-GEL fracture surface (scale bar = 200 μm). **(E)** Comparison of swelling ratios between SF-GEL and silk. **(F)** Rheological strain sweep curve of SF-GEL. **(G)** Rheological frequency sweep curve of SF-GEL. **(H)** Rheological time sweep curve of SF-GEL.

SF-GEL, as a biomaterial with excellent biocompatibility, has demonstrated significant application value in the field of drug delivery in recent years. Preparation of lyophilized SF powder (Zhao et al., 2021)from silk was performed according to the steps outlined in Figure 2A. The lyophilized SF powder was then reconstituted to obtain SF-aq. Gelation of SF-aq was subsequently carried out using the HRP/H_2_O_2_ system to prepare SF-GEL. As shown in Figure 2B, SF-aq can be transformed from a liquid into a non-flowable solid within 12 min (McGill et al., 2020). This rapid phase transition from liquid to solid holds good application prospects for diseases requiring open surgical procedures, such as osteoarthritis. Based on this, the mixture of HRP/H_2_O_2_ and SF-aq was successfully delivered physically through a medical syringe needle. Gelation occurs *in situ* at the target site, forming SF-GEL with the desired shape (Figure 2C) (Chen et al., 2024). Studies indicate that active oxygen free radicals are generated through the catalytic decomposition of H_2_O_2_ by HRP. These radicals oxidize tyrosine residues in silk fibroin (SF), resulting in the formation of tyrosine radicals. Adjacent tyrosine radicals undergo covalent coupling to form dityrosine bonds. Ultimately, a covalent cross-linked network with dityrosine bonds as nodes is constructed between SF molecules, achieving stable cross-linking of biomacromolecules (Wang et al., n.d.). SF-GEL can be injected into the lesion site via a minimally invasive approach and achieves *in situ* gelation, thereby reducing tissue damage associated with open surgery.

The microscopic morphology of SF-GEL was further characterized by SEM, with results shown in Figure 2D. A uniformly distributed honeycomb-like pore structure was observed on the fracture surface of SF-GEL. The pore diameters primarily ranged from 50 to 200 μm, exhibiting a typical three-dimensional porous network structure. The observations indicate that SF-GEL possesses a loose and porous structure (Kim et al., 2018), which not only allows tighter integration with tissues after water absorption and swelling but also provides channels for drug delivery, holding crucial significance for potential biomedical applications. Based on this, the swelling properties of SF-GEL were further characterized using the gravimetric method. Experimental results in Figure 2E show that SF-GEL exhibited a swelling ratio as high as 546%, whereas silk material demonstrated a swelling ratio of only 95%. This performance difference is mainly attributed to the distinct microstructures: SF-GEL forms a three-dimensional porous network through cross-linking, with its high internal porosity providing ample space for water penetration and storage. In contrast, natural silk is predominantly composed of β-sheet structures, featuring densely packed molecules and low porosity, resulting in limited water absorption capacity. The highly swellable SF-GEL can mimic the physical properties of the extracellular matrix, avoiding foreign body reactions (Yu et al., 2021).

Furthermore, in inflammatory treatments requiring filling and repair, such as osteoarthritis, the mechanical properties of implanted materials are critical for resisting tissue compression (Bryant, 2016). Based on this, the rheological properties of SF-GEL were further investigated by rotational shear rheometry, with results presented in Figures 2F–H. The storage modulus (G′) of SF-GEL consistently exceeded the loss modulus (G″), exhibiting elastic-dominant behavior without a viscoelastic state transition. This characteristic rheological response confirms the successful construction of a three-dimensional network structure in SF-GEL. This network effectively encapsulates anti-inflammatory active ingredients while enabling drug delivery through pore channels (Oral et al., 2020).

### 3.2 Synthesis and characterization of PES and PNES

**FIGURE 3:**
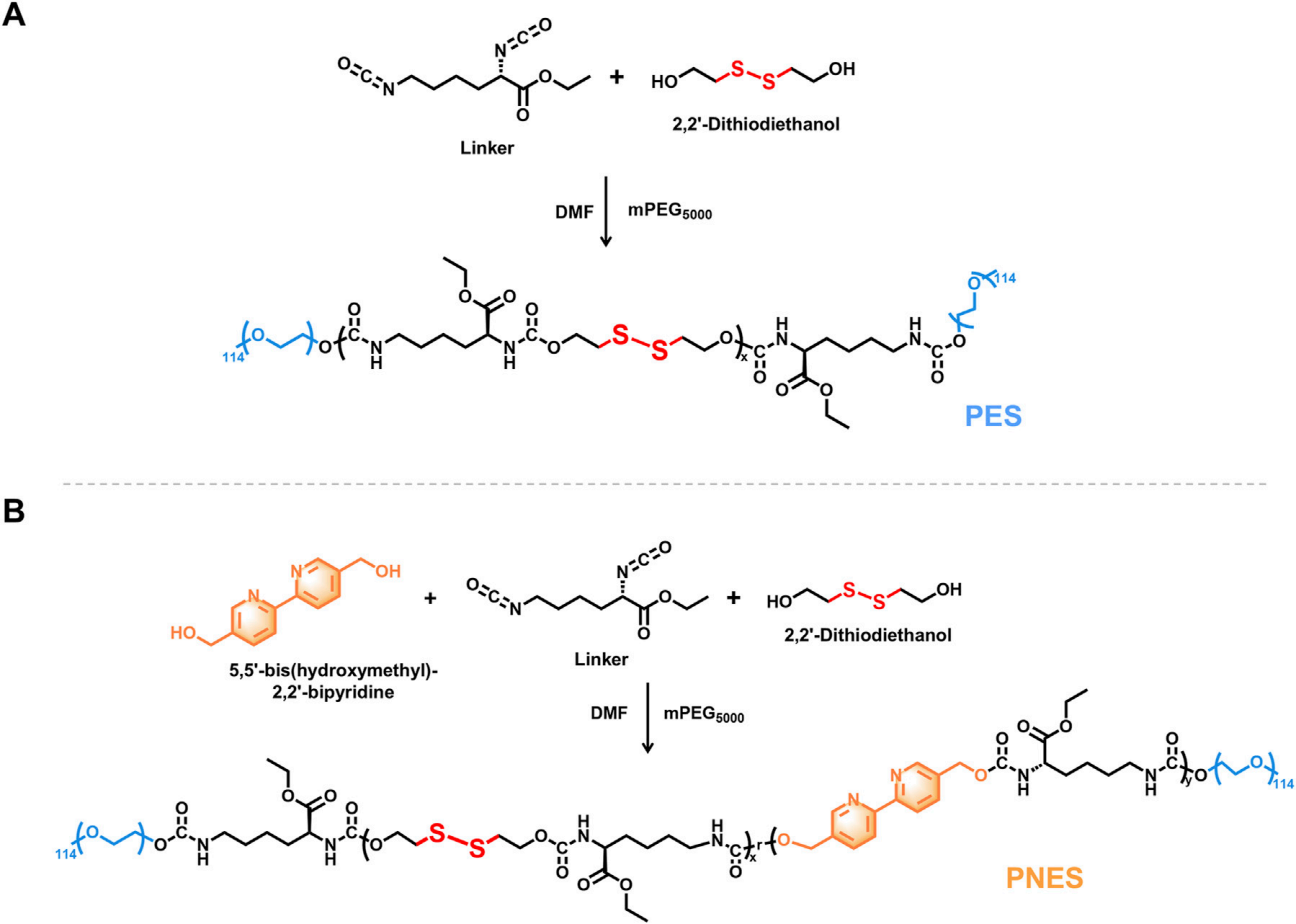
Synthetic route of PES and PNES. **(A)** Synthetic route of PES. **(B)** Synthetic route of PNES.

Although SF-GEL possesses favorable mechanical properties, it is difficult to load water-insoluble drugs (e.g., CUR) and lacks responsive drug release capability. To address this, the nanoparticle NP@PNES-CUR was constructed, combining water solubility with GSH-responsive drug release functionality. Firstly, as illustrated in Figure 3A, 2,2′-Dithiodiethanol and Linker were polymerized, followed by capping with mPEG_5000_, to synthesize PES. The PES backbone is rich in disulfide bonds, imparting GSH-responsive cleavage characteristics. The PEG segments at the chain termini, composed of repeating ethylene glycol units (-O-CH_2_-CH_2_-), confer hydrophilicity to both ends of PES.

Subsequently, to enhance the drug loading efficiency of the polymer for conjugated system drugs (e.g., CUR), PNES was synthesized by introducing 2,2′-Bipyridine-5,5′-dimethanol (Figure 3B). The conjugated system in the bipyridyl groups of PNES promotes uniform electron distribution, reducing the local polarity of PNES (Chen et al., 2022) and increasing the hydrophobicity of its mid-section. Consequently, this acquired amphiphilic nature provides a theoretical basis for the self-assembly of PNES with the hydrophobic drug CUR in aqueous solution to form NP@PNES-CUR.

**FIGURE 4:**
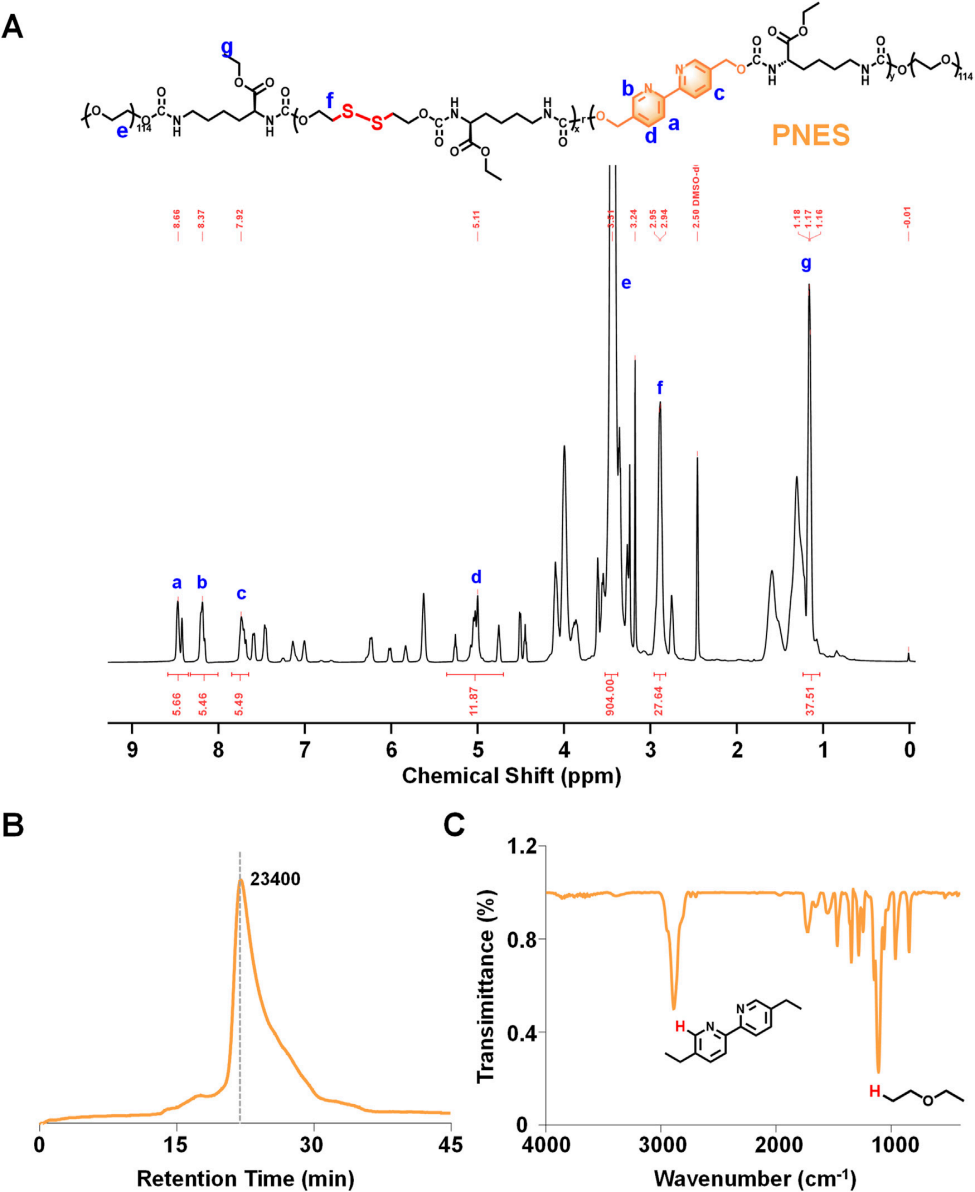
Characterization of PNES. **(A)** ^1^H NMR results of PNES. **(B)** GPC results of PNES. **(C)** FTIR results of PNES.

The ^1^H NMR spectrum of the synthesized PNES, calibrated using the solvent peak of deuterated dimethyl sulfoxide at 2.50 ppm, clearly displayed the characteristic absorption peaks of the target product (Figure 4A). Hydrogen atoms ortho, meta, and para to the nitrogen atoms in the bipyridyl ring structure exhibited characteristic chemical shifts. The meta-hydrogen peak was labeled as peak*a*at approximately 8.66 ppm. The ortho-hydrogen peak, positioned at a relatively higher field of approximately 8.37 ppm, was labeled as peak*b*. The para-hydrogen peak appeared at a relatively lower field of approximately 7.92 ppm and was labeled as peak*c*. Hydrogen atoms on the carbon linked to the bipyridyl group were labeled as peak*d*at approximately 5.11 ppm. The strong characteristic peak of hydrogen atoms in the repeating units of polyethylene glycol (PEG) was observed at 3.51 ppm and labeled as peak*e*. Hydrogen atoms on carbons adjacent to the disulfide bond were labeled as peak*f*at 2.94 ppm. The methyl proton signal of the terminal isocyanate group appeared near 1.2 ppm and was labeled as peak*g*. Assignment of the chemical shifts for all characteristic peaks was observed to be consistent with the target molecular structure, confirming that the synthesized product was indeed the target compound PNES.

Gel permeation chromatography (GPC) results of PNES indicated a weight-average molecular weight of approximately 23,400 (Figure 4B). A single peak shape was observed, demonstrating good dispersity of PNES. Fourier-transform infrared spectroscopy (FTIR) results of PNES revealed a characteristic C-H peak of bipyridyl at 2,900 cm^-1^ (Figure 4C), and characteristic ether functional group peaks of PEG within the range of 1,275–1,020 cm^-1^. Combined with the aforementioned ^1^H NMR results, synthesis of the target product PNES was confirmed.

### 3.3 Preparation and characterization of NP@PNES-CUR

**FIGURE 5:**
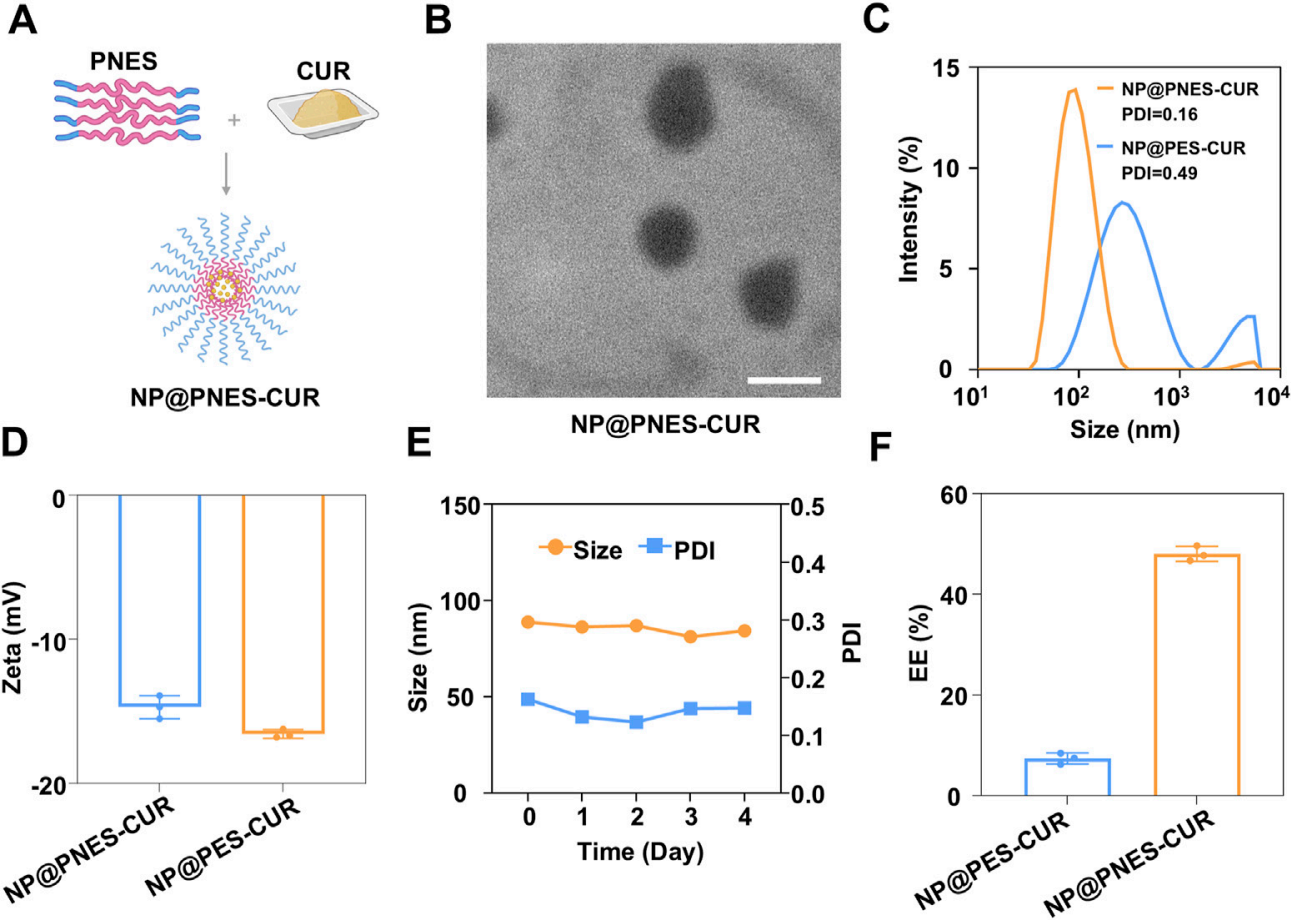
Preparation and characterization of nanoparticles. **(A)** Preparation process of NP@PNES-CUR. **(B)** TEM image of NP@PNES-CUR (scale bar = 100 nm). **(C)** Particle size distribution of NP@PNES-CUR and NP@PES-CUR. **(D)** Zeta potential of NP@PNES-CUR and NP@PES-CUR. **(E)** Stability of NP@PNES-CUR over 5 days. **(F)** Comparison of encapsulation efficiency between NP@PNES-CUR and NP@PES-CUR.

Research indicates that within the inflammatory microenvironment, cells increase GSH synthesis by activating the antioxidant defense system (Vasamsetti et al., 2016). Therefore, PES and PNES were utilized separately to encapsulate CUR. GSH-sensitive drug-loaded nano-delivery systems, NP@PES-CUR and NP@PNES-CUR, were successfully constructed via the nanoprecipitation method (Figure 5A).

NP@PNES-CUR was observed by transmission electron microscopy (TEM). A uniform spherical morphology was observed for NP@PNES-CUR, with an average diameter of approximately 111.2 nm (Figure 5B). The hydrodynamic diameter of NP@PNES-CUR was further measured by dynamic light scattering (DLS) as 87.6 nm (Figure 5C). NP@PNES-CUR exhibited a PDI of 0.16 (Figure 5C), indicating the successful preparation of nanoparticles with uniform size. The uniformity was significantly superior to that of NP@PES-CUR (PDI = 0.49). The surface zeta potential values of NP@PES-CUR and NP@PNES-CUR were further measured as −16.6 mV and −14.7 mV, respectively (Figure 5D).

To further investigate the stability of NP@PNES-CUR, changes in particle size at 4°C were monitored by DLS. Results indicated that the particle size variation of NP@PNES-CUR was less than ±5% over 5 days, and the PDI was maintained within the range of 0.12–0.16 (Figure 5E). This demonstrates the good stability of NP@PNES-CUR, which is beneficial for its storage and transportation. In contrast, NP@PES-CUR exhibited a PDI of 0.49, indicating significantly lower uniformity compared to NP@PNES-CUR.

The encapsulation efficiencies of NP@PES-CUR and NP@PNES-CUR were further investigated by quantitative HPLC testing. An encapsulation efficiency of 47.7% for CUR was achieved by NP@PNES-CUR (Figure 5F). Under identical preparation and characterization conditions, the CUR encapsulation efficiency of the PES carrier was measured at only 7.5%, a value nearly six times lower than that of NP@PNES-CUR. The significant difference between the two sets of experimental data directly confirms the decisive role of 2,2′-Bipyridine-5,5′-dimethanol in enhancing the CUR loading performance.

In summary, the rigid planar configuration of 2,2′-Bipyridine-5,5′-dimethanol effectively enhances π-π stacking interactions between polymer molecular chains. Simultaneously, its electron-rich aromatic ring structure enables the formation of specific intermolecular interactions with the phenolic hydroxyl and ketone groups in CUR molecules (Wu et al., 2006). These characteristics synergistically improve the affinity of PNES for CUR and encapsulation stability (Chelike et al., 2020). The experimental data validate the effectiveness of the conjugated structure design.

**FIGURE 6:**
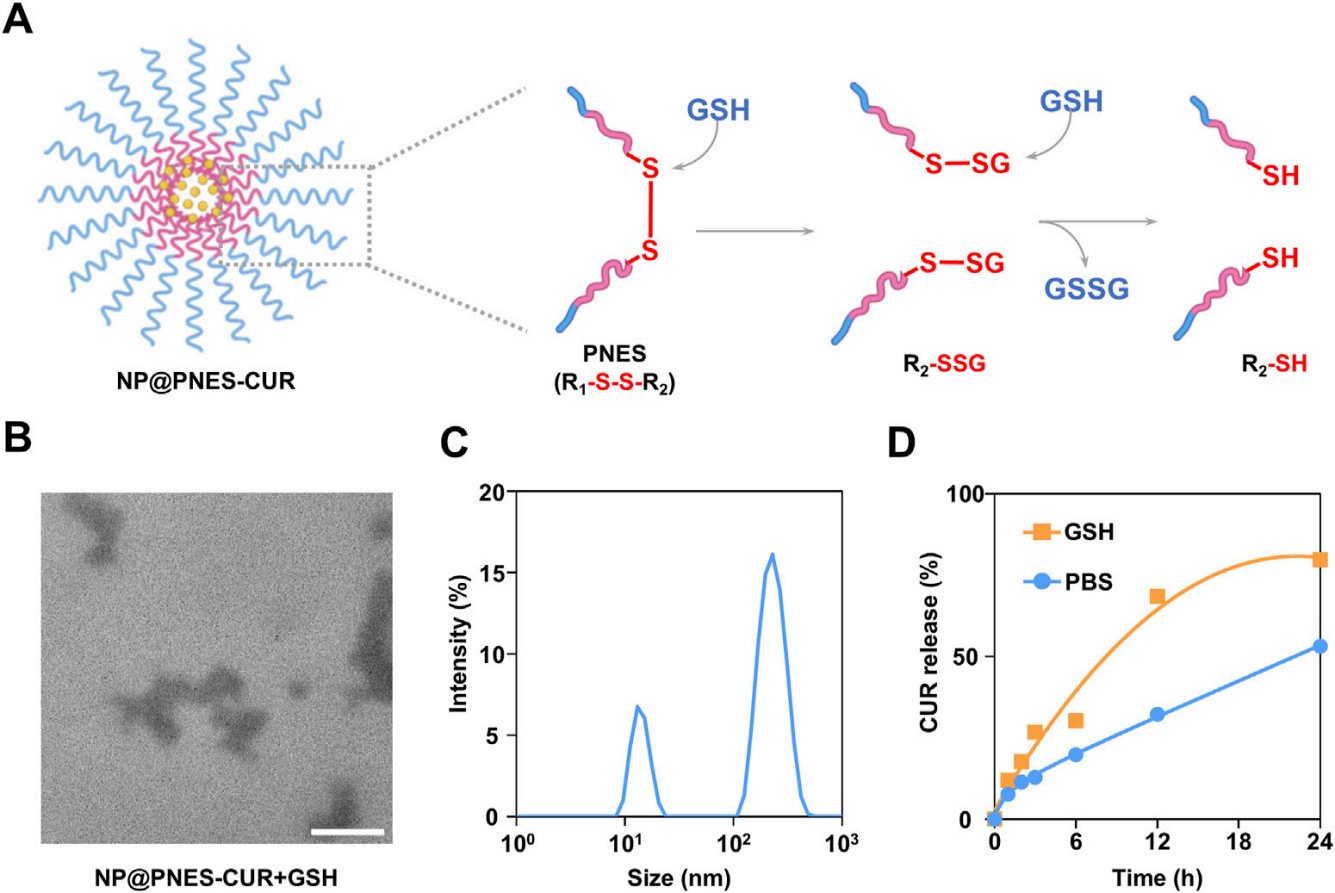
CUR release, dissociation, and characterization of NP@PNES-CUR. **(A)** Schematic illustration of the GSH-responsive dissociation of NP@PNES-CUR. **(B)** TEM image of dissociated NP@PNES-CUR (scale bar = 100 nm). **(C)** Particle size distribution of dissociated NP@PNES-CUR. **(D)** CUR release profile of NP@PNES-CUR in the GSH environment.

Following the characterization of the structure and encapsulation capability of NP@PNES-CUR, its ability to dissociate and release CUR was further investigated. CUR release from NP@PNES-CUR primarily occurs through disulfide bond cleavage, with the mechanism illustrated in Figure 6A. Cleavage of disulfide bonds is achieved by highly expressed GSH in the inflammatory environment via a thiol-disulfide exchange reaction. Studies indicate that the thiol group (-SH) of GSH is partially deprotonated to the thiolate anion (-S^-^) at physiological pH, possessing strong nucleophilicity. This anion attacks one sulfur atom of the disulfide bond, leading to cleavage and the formation of a mixed disulfide intermediate (-SSG). Subsequently, a second GSH molecule attacks the sulfur atom in the mixed disulfide, releasing a free thiol (-SH). Concurrently, two GSH molecules are connected by a disulfide bond to form oxidized glutathione (GSSG), resulting in disulfide bond cleavage (Fu et al., 2022).

To investigate the dissociation state of NP@PNES-CUR, dissociated NP@PNES-CUR was observed by TEM. It was found that the originally circular NP@PNES-CUR successfully dissociated in the GSH environment (Figure 6B). Analysis of dissociated NP@PNES-CUR by DLS revealed a PDI exceeding 0.70 (Figure 6C), which differs significantly from the PDI (0.16) measured prior to dissociation. Furthermore, DLS results showed peaks corresponding to particles with vastly different sizes. Based on this, it was concluded that the originally uniform and stable NP@PNES-CUR successfully dissociated in the GSH environment, consistent with TEM observations.

The cumulative release of CUR from NP@PNES-CUR in a GSH environment was further monitored by HPLC. For this purpose, sustained CUR release from disulfide-containing NP@PNES-CUR was tested separately in PBS and in a GSH environment simulating inflammation. As shown in Figure 6D, only 53.2% of CUR was released from NP@PNES-CUR within 24 h in PBS. In contrast, a CUR release rate of 79.8% was achieved within 24 h in the GSH environment simulating inflammation, indicating that NP@PNES-CUR responds to the GSH environment by releasing CUR.

### 3.4 Preparation and characterization of GEL-PNES-CUR

Despite NP@PNES-CUR demonstrating functionalities such as specific CUR release, the issue of non-specific distribution still constrains its clinical translation efficiency, making it difficult for CUR to stably accumulate in inflammatory regions over the long term. Simultaneously, existing nano-systems lack the mechanical properties to adapt to complex mechanical environments, failing to meet the mechanical demands placed on carrier materials by joint movement during the treatment of diseases such as osteoarthritis. To address these bottlenecks, a composite system of SF-GEL loaded with NP@PNES-CUR was constructed. The inherent biocompatibility of SF and the tunable mechanical properties of SF-GEL were utilized. This approach preserved the GSH environment-responsive CUR release capability of NP@PNES-CUR while endowing the carrier material with mechanical adaptability matching the physiological environment. The biocompatibility of the SF-GEL composite system loaded with NP@PNES-CUR was also investigated. Therefore, NP@PNES-CUR was blended with SF-aq and gelated using the HRP/H_2_O_2_ system to prepare SF-GEL loaded with NP@PNES-CUR, designated as GEL-PNES-CUR.

**FIGURE 7:**
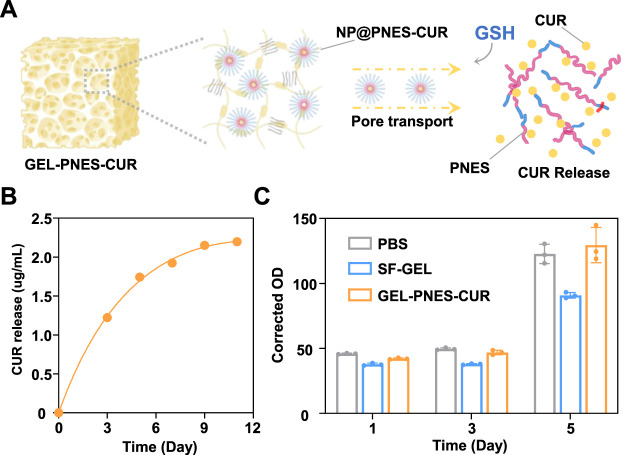
Characterization of GEL-PNES-CUR. **(A)** Schematic illustration of the CUR release process from GEL-PNES-CUR. **(B)** CUR release profile of GEL-PNES-CUR. **(C)** MTT assay of GEL-PNES-CUR.

To investigate the CUR release profile of GEL-PNES-CUR in an environment simulating inflammation, CUR release was studied in a GSH environment mimicking the inflammatory microenvironment. The CUR release process is illustrated in Figure 7A. Analysis by HPLC revealed that GEL-PNES-CUR exhibited a characteristic slow release of CUR over the experimental period. By day 3, the CUR concentration in the dialysate outside the dialysis bag released from GEL-PNES-CUR reached 1.2 μg/mL, and by day 11, the cumulative CUR concentration reached 2 μg/mL (Figure 7B).

Based on this, analysis indicated that the GSH-responsive release characteristics of GEL-PNES-CUR align with the pathological microenvironment features of inflammation. Elevated GSH levels in the environment will trigger CUR release from NP@PNES-CUR, while the sustained release behavior under normal physiological conditions can prolong the action time of CUR.

GEL-PNES-CUR forms a dense three-dimensional cross-linked network through secondary structures such as β-sheets. The pore size and distribution within GEL-PNES-CUR directly influence the GSH infiltration rate and CUR diffusion rate, where smaller pores may slow down the free diffusion of CUR (Wang L. et al., 2025). Furthermore, upon water absorption and swelling, the pores within GEL-PNES-CUR may potentially enlarge and accelerate CUR diffusion. However, the swelling process itself requires time, and the initial swelling phase may suppress excessively rapid CUR release.

To further systematically evaluate the cytocompatibility of GEL-PNES-CUR, quantitative cytotoxic analysis was performed using the 3-(4,5-dimethylthiazol-2-yl)-2,5-diphenyltetrazolium bromide (MTT) method. Three control groups were established: SF-GEL group, GEL-PNES-CUR group, and PBS blank control group, co-cultured with rat fibroblasts (Rat-FB) as the model. As shown in Figure 7C, initial detection after 24 h of Rat-FB culture revealed absorbance values of 42.1 ± 0.7 for the SF-GEL group and 37.7 ± 1.1 for the GEL-PNES-CUR group. These values showed no statistically significant difference (p > 0.05) compared to the PBS control group (45.9 ± 0.5). This result indicates that neither material exhibited cytotoxic effects during the initial culture period. Notably, differentiated proliferation trends were observed upon continued culture of Rat-FB to day 5. The absorbance value for the SF-GEL group was maintained at 90.9 ± 2.4, significantly lower than that of the PBS control group (122.7 ± 7.8, p < 0.05). In contrast, the GEL-PNES-CUR group reached 129.5 ± 15.4, showing no statistically significant difference compared to the control group (p = 0.083). This phenomenon confirms the excellent inherent biocompatibility of the SF-GEL matrix itself, and demonstrates that the introduction of NP@PNES-CUR did not compromise material safety. Furthermore, GEL-PNES-CUR promoted Rat-FB cell proliferation through the controlled release of CUR.

Mechanistic studies indicate that the enhanced bioactivity of GEL-PNES-CUR originates from its unique drug delivery properties, where the porous scaffold structure ensures sustained CUR release. CUR exerts its anti-inflammatory effects by inhibiting the activation of inflammatory signaling pathways such as nuclear factor κB (NF-κB), thereby reducing the production of pro-inflammatory cytokines (e.g., TNF-α, IL-6). Simultaneously, it downregulates cyclooxygenase-2 (COX-2) and reactive oxygen species (ROS) production, blocking the release of inflammatory mediator (Yang et al., 2017). Throughout the testing period, cell viability in the experimental group was maintained above 70%, meeting the requirements of the ISO 10993–5 standard for medical device biocompatibility (Kovrlija et al., 2024). The comprehensive experimental data demonstrate that GEL-PNES-CUR enables sustained delivery of CUR and maintains normal fibroblast proliferation.

## 4 Conclusion

The clinical application of CUR is severely limited due to its inherent poor water solubility and low bioavailability. To address this challenge, a GSH-responsive drug-loaded composite hydrogel system, GEL-PNES-CUR, was successfully constructed in this study. GEL-PNES-CUR integrates excellent mechanical adaptability with controllable drug release functionality. Firstly, the constructed SF-GEL demonstrated good injectability, a remarkably high swelling ratio (546%), and excellent rheological properties (G′ up to 10,000 Pa). This enables minimally invasive *in situ* filling of lesions and provides necessary mechanical support. Secondly, GSH-responsive polymeric polymers, PES and PNES, were designed and synthesized. Nanoparticles loaded with CUR, NP@PES-CUR and NP@PNES-CUR, were prepared using the nanoprecipitation method. Characterization results revealed that NP@PNES-CUR possessed uniform particle size (87.6 nm) and a low polydispersity index (PDI = 0.16), significantly superior to NP@PES-CUR (PDI = 0.49). More importantly, an encapsulation efficiency of CUR as high as 47.7% was achieved for NP@PNES-CUR, markedly higher than that for NP@PES-CUR (7.5%). The experimental results fully demonstrated the effectiveness of the bipyridyl conjugated structure design in enhancing drug loading efficiency. *In vitro* release experiments further confirmed that NP@PNES-CUR could respond to a high GSH environment (simulating the inflammatory microenvironment), achieving 79.8% CUR release within 24 h. Ultimately, the composite hydrogel GEL-PNES-CUR was successfully prepared by loading NP@PNES-CUR into an SF-aq solution followed by gelation using the HRP/H_2_O_2_ system. This composite system exhibited sustained slow-release characteristics of CUR in a simulated inflammatory microenvironment, with a release period extending up to 11 days. Good biocompatibility of GEL-PNES-CUR was further confirmed by MTT cytotoxicity assays.

In summary, this study innovatively combined the excellent mechanical adaptability of SF-GEL with the controlled drug release functionality of the GSH-responsive nanoparticle (NP@PNES-CUR), successfully developing the GEL-PNES-CUR composite delivery system. This provides a novel multifunctional material possessing both environmental responsiveness and dynamic mechanical stability.

## Data Availability

The raw data supporting the conclusions of this article will be made available by the authors, without undue reservation.
